# “Life gives you a lemon, you make lemonade”: a qualitative study of identity among young male adult cancer survivors

**DOI:** 10.1007/s00432-025-06317-4

**Published:** 2025-10-09

**Authors:** Glenn F. Flecther, May Aa. Hauken

**Affiliations:** Faculty of Psychology, Centre for Crisis Psychology, University of Bergen, Jekteviksbakken 31, 5006 Bergen, Norway

**Keywords:** Male YACSs, Identity, Cancer survivor, Identity development, Agency and communion, Qualitative study

## Abstract

**Purpose:**

This study aimed to explore how male young adult cancer survivors (YACSs), aged 18–39, reconstruct and make sense of their identity following the completion of cancer treatment by answering the research question: “How do male YACS perceive and interpret changes in their identity following cancer treatment?

**Methods:**

A qualitative approach with an interpretive descriptive design was employed. Drawing on the theoretical framework of Agency and Communion, individual semi-structured interviews were conducted with 12 male YACS. The transcribed interviews were analyzed using Systematic Text Condensation.

**Results:**

The findings indicate that the identities of male YACS underwent significantly transformation following cancer treatment. The overarching theme “*A changed and matured identity*” was identified and elaborated by three main themes (1) “*I feel like an old man*”, (2) “*My values and perspectives have changed*”*,* and (3) “*I have some advice to share*”. Participants reported impaired physical, cognitive and social capacities, which contributed to shift in their sense of self. These changes required them to adapt to new life circumstances, often affecting their ability to pursue age-normative goals critical to identity development.

**Conclusion:**

The findings indicate that the participants’ pre-cancer identities, which emphasized agentic qualities over communal ones, were significantly altered. Post-treatment, they experienced a reorientation of values and priorities, shifting from an agentic to more communal self-perception contributing to the development of a more mature identity and a revised outlook on life. These findings may serve as a foundational basis for future research and to inform the development of clinical practices.

**Supplementary Information:**

The online version contains supplementary material available at 10.1007/s00432-025-06317-4.

## Introduction

In Norway, cancer in young adulthood (ages18–39) accounts for only 2.8% of new cases in males (Cancer Registry of Norway 2025). Despite its rarity, cancer in this age group presents distinct biology, pathological, and psychosocial challenges. Young adult cancer survivors (YACS) often undergo intensive treatment, face lower survival rates, and remain underrepresented in research compared to pediatric and older adult populations (Bleyer et al. 2008; Close et al. 2019; Trama et al. 2018).

Young adulthood typically involves transitions in education, relationships, career, and financial independence, making cancer an especially disruptive burden (Arnett 2000; Hauken et al. 2019; Hutteman et al. 2014; Patterson et al. 2015). Due to multimodal treatments, YACSs are at increased risk for physical late effects across organ systems (Baker et al. 2017; Bhuller et al. 2016) as well as psychological distress, including anxiety, depression, fear of recurrence, and sexual dysfunction (Jones et al. 2011; Rincones et al. 2021). They also report strained relationships and social support (Benedict et al. 2018; Hauken and Larsen 2019), career setbacks, financial hardship (Blanch-Hartigan and Kinel 2018; Stone et al. 2017; Vetsch et al. 2018), and unmet follow-up needs (Baudry et al. 2024; Bibby et al. 2017).

YACS challenges highly intersect with identity development, a central task in young adulthood (Bagautdinova et al. 2024; Barbot et al. 2022; Kumar and Schapira 2013). Identity is shaped by interactions with peers, family, culture, and personal experiences (Erikson 1968; Rogers 2018). Agency and Communion theory offers a powerful lens for understanding identity formation, particularly in how individuals perceive themselves and relate to others. In this theory, identity development distinguishes between agentic traits (e.g. autonomy, competence, control and goal achievement) and communal traits (e.g., connectedness, belonging and social affiliation) (Abele and Wojciszke 2019; Chan et al. 2019). Cancer-related disruptions can challenge both domains, affecting self-perception and identity formation (Benedict et al. 2018; Blanch-Hartigan and Kinel 2018; Hauken and Larsen 2019; Stone et al. 2017; Vetsch et al. 2018).

Although identity among adult cancer survivors has been explored, little is known about YACS specifically. Research indicates that adult survivors may adopt various post-treatment identities—such as “patient,” “victim,” or “survivor”—each with distinct psychological implications (Cheung and Delfabbro 2016; Park et al. 2009). Research also shows that identity disruptions may stem from bodily changes, cognitive decline, and social isolation, all of which can impair continuity of self (Kuswanto et al. 2018; Martino et al. 2024). While adopting a “survivor” identity, this is linked to optimism and post-traumatic growth (Cheung and Delfabbro 2016; Morris et al. 2014; Park et al. 2009) and many struggle to return to pre-cancer identities (Jones et al. 2011). Men, in particular, may resist the survivor label to avoid being defined by illness (Dalton et al. 2021; Morris et al. 2014), and issues like erectile dysfunction can deeply affect masculine identity (Dalton et al. 2021; Zaider et al. 2012).

The limited research on identity in YACS, often predominantly in females, suggest that YACSs strive for normalcy and independence, while functional impairments may reinforce feelings of difference from peers (Jones et al. 2011; Kumar and Schapira 2013). Positive identity development in YACS seem to be associated with good health and meaning making, while poor health and trauma correlate with identity confusion (Prikken et al. 2021; Vanderhaegen et al. 2023). Research also indicates that confronting existential challenges may foster personal growth and value shifts (Jones et al. 2011; Levin-Dagan and Hamama 2024). In YACS’ experiences of own identity, infertility is a major concern, particularly for men. This concern is linked to emotional distress, feelings of inadequacy, and social withdrawal (Dax et al. 2022; Ussher and Perz 2019). While both genders experience identity shifts, women tend to emphasize social identity and communal values, whereas men focus on autonomy and achievement (Sczesny et al. 2019). Male YACS face distinct identity challenges, including stigma in rare diagnoses like male breast cancer (Dalton et al. 2021), and increased suicide risk among unmarried longterm survivors (Zhou et al. 2019). However, more research on how male YACS reconstruct and make sense of their identity following cancer treatment is warranted to develop tailored follow-up services. Consequently, this study aims to answer the research question: “How do male YACS perceive and interpret changes in their identity following cancer treatment?".

## Methods

This qualitative study employed an interpretive descriptive design, which is an indictive, constructivist approach aiming to generate clinically relevant knowledge (Thorne et al.^1997^). Through an iterative and dynamic process between the data and the researchers’ interpretations to explore complex phenomena, such as identity changes in male YACS post-treatment.

### Recruitment and participants

Purposeful sampling was used to recruit participants (Suri 2011) based on the following inclusion criteria: (1) males aged 18–39, (2) diagnosed with cancer after age 18, (3) Norwegian-speaking, and (4) at least six months post-treatment. Exclusion criteria included: (1) coexisting chronic somatic or mental illness affecting self-perception, and (2) ongoing cancer treatment.

Recruitment was conducted via the Norwegian Cancer Society and the Young Cancer Society. The Cancer Society promoted the study through its website and posters at Beacon Centres, while the Young Cancer Society distributed information via email and social media (Facebook, Instagram).

Of 16 respondents responding, 12 met the inclusion criteria and were enrolled. Participant demographics and clinical characteristics are presented in Table 1.

**Table 1:** Demographic and medical variables of the participants

	N	Mean	SD/Range
Age, years	12	31	5.2/23–39
Civil status			
Married	3	25%	
In a relationship	6	50%	
Single	3	25%	
Highest education			
Senior Highschool	3	25%	
University/College	9	75%	
Work/study			
In work	9	75%	
Student	1	8.3%	
Treatment			
Surgery	7	58.3%	
Chemotherapy	10	83.3%	
Radiation Therapy	6	50%	
Immune Therapy	1	8.3%	
Cancer diagnosis			
Testes	2	16.7%	
Astrocytma	1	8.3%	
Lymphoma	3	25%	
Oral Cancer	1	8.3%	
Leukemia	2	16.7%	
Brain Cancer	1	8.3%	
Sarcoma	3	25%	
Months since the end of treatment	12	79.1%	77.2/27–261

N = Number of informants, SD = Standard deviation

### Data collection

Data was collected through individual; in-depth interviews conducted digitally via Microsoft Teams by the first author. Before each interview, the researcher introduced himself and reiterated the interview’s purpose and procedures. Except for three participants previously encountered through the Young Cancer Society, the researcher had no prior relationship with the informants; all interviews followed the same protocol.

A semi-structured interview guide, grounded in the identity framework of agency and communion, was used. The opening question—“Can you please describe how you experience your identity after completing cancer treatment?”. Informants were encouraged to freely outline their experiences, with follow-up questions related to social roles, body image, life goals, confidence, and coping. Interviews were audio-recorded, lasted 29–73 min, and were supplemented with field notes capturing non-verbal cues and emotional expressions. Data saturation was reached after 12 interviews. All recordings were transcribed verbatim by the first author, with identifying details removed to ensure anonymity. No repeat interviews were conducted, and participants did not review transcripts or provide feedback on findings. The final dataset comprised 170 pages of transcribed data.

### Data analysis

Data was analyzed using Systematic Text Condensation (STC) (Malterud 2012), a four-step method well-suited to interpretive descriptive research. NVivo software supported the organization and facilitation of the analysis (Lumivero 2025). Table 2 provides an overview of the process, which is detailed below.

**Table 2:** Overview of the analyze process

**Step 1**: Total impression Process	Identified total impression		
Each author independently reviewed the transcribed interviews and then collectively discussed their overall impressions until they reached a consensus	A new state of normalcy characterized by physical and cognitive limitations Altered physical self-image Changed social life Evolved values, perspectives, and coping strategies Acceptance of a new existence Mourning the loss of one's former self		
**Step 2**: Identifying and sorting meaning units Process	Identified meaning units Code	Files	References
The researchers independently reviewed all transcripts again, coding them with meaning units. These codes were then discussed until consensus was achieved, with a focus on differences to broaden the analytical perspective	Loss and vulnerability	12	106
	Reduced physical, cognitive and 12	81 social capacities	
	Evolved values and perspectives	12	54
	Self-efficacy and self-esteem	12	52
	Changed identity	11	52
	Acceptance of a new existence	12	46
	Coping Strategies	10	42
	Altered physical self-image	10	34
	Advice	12	23
	Things take time	8	15
**Step 3**: Condensation from meaning units to themes Process	Identified content/themes		
The content of each code was discussed until the consensus was reached. These codes were then further condensed into bridging themes, which were elaborated into main themes and subthemes	Overarching theme: A changed and matured identity The analyses identified one overarching theme, three main themes, each further elaborated with two accompanying subthemes		
**Step 4**: Synthesizing the findings Process	Findings		
The thematic content was synthesized and integrated into an interpretation of the findings	The themes were summarized and illustrated with statements from the participants		

Files = (n) participants who referenced the meaning unit

References = (n) instances of the meaning unit being mentioned

During the first step, the authors independently reviewed the transcripts to gain an initial understanding of the thematic content. They then discussed and agreed upon six initial themes (Step 1 in Table 2). In the second step, transcripts were re-read line by line to identify meaning units. These were then discussed and grouped into 10 codes based on insights from Step 1 (Table 2, Step 2). In the third step, codes were decontextualized and analyzed independently by both researchers to ensure validity. Through discussion and comparison with the transcripts, they identified one overarching theme, three main themes, and six subthemes (Table 2, Step 3). In the final step, themes and subthemes were synthesized into conceptual descriptions, supported by participant quotes, to ensure the findings reflected the informants’ intended meaning (Table 2, Step 4). Each step of the analyses involved multiple analytical cycles until consensus was reached. To facilitate reflexivity, the authors—a male psychologist and cancer survivor and a female professor with clinical and research experience—reflected on how their backgrounds might influence interpretation (Malterud 2012; Olmos-Vega et al. 2023).

### Ethics

The Regional Committee for Ethics in Medical and Health Research (REK) reviewed the study and deemed it outside the scope of the Norwegian Act on Medical and Health Research (ref. no. 812837). The Norwegian Agency for Shared Services in Education and Research (SIKT) approved the project regarding information security, data protection, and processing (ref. no. 244519), and it was registered in the institutional research system RETTE (ID number S3700).

Participants received written and verbal information about the study, and informed consent was obtained either in writing or through recorded verbal agreement. The study followed the Declaration of Helsinki (World Medical Association 2024). Audio recordings were deleted after transcription, and anonymized transcripts were securely stored in accordance with the University of Bergen’s guidelines.

The COREQ checklist was applied in reporting this study (Attachment 1). Given the sensitivity of cancer-related topics, participants were informed they could access follow-up support from the Center for Crisis Psychology.

However, no one requested this service.

## Results

The analysis revealed that participants experienced a significant shift in identity following their cancer diagnosis and treatment. The overarching theme, **“**A changed and matured identity,” captured how cancer treatment— combined with the natural process of aging—reshaped their sense of self. As one participant put it: *“Life gives you a lemon, you make lemonade”* (Dan), reflecting a mindset of resilience and adaptation. This overarching theme was further elaborated by three main themes (1) “I feel like an old man”, (2) “My values and perspectives have changed”, and (3) “I have some advice to share.” Each was further developed through two subthemes.

These findings are summarized in Table 3 elaborated below.

**Table 3:** The study’s main findings

A changed and matured identity	
Main themes	Sub themes
“I feel like an old man”	a) “My capacity is not close to what it used to be” b) “I have lost a lot, and my future is vulnerable”
“My values and perspectives have changed”	a) “I had to adapt to my new life” b) “I have accepted my new reality”
“I have some advice to share”	a) “You are not alone—speak to someone” b) “It takes time to mend”

### Theme 1: “I feel like an old man”

The first main theme highlighted how all participants faced physical, cognitive, or social challenges after cancer treatment. These challenges included fatigue, physical limitations, and the loss of pre-cancer activities—such as sports, socializing, or working impacting their daily routines and sense of identity. As Peter shared: *“It has something to do with (…) really (…) the feeling of independence (…) I meant by feeling like an old man. An old grandpa that needs help. I had my apartment, work, and salary before becoming sick, everything was ok.”* This theme is further explored through the following two subthemes.

#### Subtheme 1a: “My capacity is not close to what it used to be”

Participants described varying degrees of physical, cognitive, and social impairments following treatment. While some were affected in just one area, others experienced limitations across all three, challenging their sense of identity—particularly in relation to competence, strength, and social connection.

Fatigue was a common late effect, contributing to difficulties with memory, concentration, and social engagement. Many avoided gatherings or physical activity due to reduced stamina. This loss of capacity affected daily functioning, making routine chores, social activities, and moderate activities challenging. As Peter shared: *“I am not nearly close to the same capacity as I had before (…) sure it is going to change, but I doubt I will ever get it. I do exercise, but that is very light. I exercised with my wife yesterday and did what she did. I was completely dead afterward. I need to pace myself, and it is more difficult for me to concentrate.”*

Consequently, participants emphasized the need to monitor their energy and plan activities accordingly. Scott explained:* “You become more aware of (…) the social part. And much more aware of one’s capacity. Am I too sick to get involved in different things? (…) I need to plan what I can and cannot do carefully. I need to be more considerate of the signals my body is telling me.”*

#### Subtheme 1b: “I have lost a lot and my future is vulnerable”

Participants described a deep sense of loss and vulnerability about the future, affecting their identity. This was tied to changes in physical appearance, work capacity, relationships, and fertility—creating a disconnect between past goals and their altered reality. Many questioned whether life would return to normal, expressing doubts about working at full capacity, maintaining social ties, or even surviving the next five years. These concerns reflected both internal struggles and fears of external judgment. As David shared:* “The uncertainty and the thought (…) will I ever experience the joy of becoming a father? The other thing is, will I ever find a partner that will accept the risk that I might not be capable of bringing forth a child?”.*

Comparisons between life before and after cancer revealed a heightened sense of vulnerability. Yet, some participants found new meaning and passions. Nicholas, who once aspired to be a professional footballer, reflected: *“Everything got turned over on its head. But I realized later (…) that it was quite ok because I have found a new passion. Snowboarding. I feel I have lost football, but that I gained (…) A bigger network of friends and another passion. I choose to look at the positive rather than focusing on the negative.”*

### Theme 2: “My values and perspectives have changed”

Cancer and its treatment led participants to reflect deeply on their lives, prompting shifts in values and identity.

Many described a renewed appreciation for everyday moments. As Ethan shared:

*“The treatment I underwent made it so I, in general, got a new perspective on life. Small things one took for granted before sickness. It may be such a simple thing as being capable of showering without any problems.”* Faced with lasting effects across all areas of life, many participants had re-evaluated their priorities, seeking new meaning and ways to adapt. These strategies are further explored in the following two subthemes.

#### Subtheme 2a: “I had to adapt to my new life”

Participants described the need to adapt to new limitations, often unable to work full-time, complete education, or pursue previous goals—deeply affecting their identity. For many, this led to a shift in values. As Michael reflected: *“Values were measured in materialistic things (…) and the pursuit of a career, I was about to say, get up and ahead. Now values are measured more by those fine moments.”*

Spending time with close family and friends became more meaningful, while participants distanced themselves from unproductive conflicts or superficial relationships. To manage cognitive challenges, most adopted practical strategies like using calendars, lists, and structured planning. These deficiencies affected their self-perception of competence as Steven shared: *“I had to let go of the stress and started using a calendar to be more organized in how I go about things. Earlier, I was not dependent on a calendar. I could remember all the important dates back and forth in time (…) It is not like that anymore.”*

#### Subtheme 2b: “I have accepted my new reality”

Acceptance emerged as a key part of survivorship. Most participants described coming to terms with their new reality as essential to rebuilding identity. While they were eager to pursue new goals, patience was often necessary—and frustrating. As Alfred shared: *“I have learned to be patient and realize it takes time. Slow and steady, as I have been told, it is quite frustrating. I really want to get started (…) I just have to realize that this is the reality, and what I can do about it is limited. I can take small steps and work myself back.”*

Many participants struggled with late effects and loss, making it important to let go of old standards and avoid self-comparisons. Rebuilding self-worth meant setting new, realistic expectations aligned with their current capacities, adjusting their identity to new capacities.

In contrast, a few participants felt less impacted by treatment and didn’t need to adjust their identity as much. Still, they gained a deeper awareness of vulnerability. As Bruce noted: *“My life did not change much, so I did not really have to change my identity. But suppose you are a person who is capable of much and becoming an individual who is capable of less. In that case, it is a different process.”*

### Theme 3: “I have some advice to share”

Cancer was described as a life-altering experience that prompted deep reflection and the development of coping strategies. Participants often felt time moved slowly and were confronted with complex thoughts. Many expressed a desire to support other men facing similar challenges by sharing advice on recovery and identity. As Jimmy advised: *“Take one step at a time and be aware that things have changed. Try to set the list right. Be extra kind to yourself and do not set too high standards. Try to feel mastery even if it’s just stepping outside to catch fresh air. Never give up; one day shall be another.”* Two subthemes emerged, detailing strategies for coping with survivorship as outlined below.

#### Subtheme 3a: “You are not alone—speak to someone”

Participants emphasized the emotional toll of cancer and the importance of connecting with others who shared similar experiences. Talking with fellow male survivors offered a sense of validation and belonging that others couldn’t provide. As Tony shared: *“Talk to someone. Try to express your feelings. You are not alone is a big one. I have been in situations where I have spoken to other male young adults who have had cancer. Talking with someone (…) makes it feel less unjust and knowing others have felt the same way.”*

Many participants expressed a loss of affiliation with earlier groups, affecting their self-perception and identity. Conversations with other male cancer survivors provided unique support that friends, family, colleagues, and partners could not, creating a new identity category “us who have lived through cancer and those who have not”.

Several also described post-treatment stress, anxiety, and intrusive thoughts that hindered recovery. Seeking professional help was seen as essential. David explained: *“The thing that helped me the most on my journey in letting go of the disease was taking the step to speak to a psychologist about it. Even though it was not a psychologist specialized in cancer, it did help to talk about it, the painful feelings.”*

Despite these struggles, some participants reported increased self-efficacy and a renewed sense of coping in other areas of life.

#### Subtheme 3b: “It takes time to mend”

Participants emphasized the importance of not rushing back to normal life. Some felt pressured—by themselves or their general practitioners—to return to work too soon, which hindered their recovery and affected their sense of identity. Instead, they advised taking time to reflect, heal, and set new, realistic goals. As Ethan shared: *“Do not stress with rushing and getting back too quickly. Spend this time thinking about your life and what you would like to do. Try to do something meaningful.”*

Recovery was described as a long process—often taking months or even years. Steven reflected:

*“It can take time before the turning point appears, but suddenly opportunities open up, and then in some weird way you get a confidence boost.”* Though prolonged recovery could bring feelings of emptiness or hopelessness, participants stressed the importance of patience, hope, and focusing on small, positive steps forward.

## Discussion

This study explored how male YACSs perceive their identity after treatment. Findings indicate that cancer prompted a transformation marked by increased maturity and a metaphorical sense of aging. The following sections discuss the key results.

### “I feel like an old man”

A key finding is that participants experienced impaired social, cognitive, and physical capacities post-treatment, which significantly impacted their identity. Fatigue and cognitive impairments were especially disruptive to work, education, social life, and daily functioning, which are core areas for achieving early adult milestones like career, independence, and family formation (Arnett 2000; Hutteman et al. 2014).

Most participants compared their pre- and post-cancer selves, striving for a sense of normalcy—a common theme among YACSs and consistent with prior research (Adamsen et al. 2009; Kumar and Schapira 2013). This shift reflects a gap between their current state and their ideal identity. Through the lens of Agency and Communion theory, diminished agentic traits—such as competence, independence, and assertiveness—contributed to this identity disruption (Chan et al. 2019).

It is well documented that cancer treatment may alter YACSs’ self-perception, disrupting their life narrative and sense of continuity (Baker et al. 2017; Hauken et al. 2013; Patterson et al. 2015), similar to findings in older populations where cancer is linked to identity loss (Clarke et al. 2011).

Infertility emerged as a major concern. Participants expressed uncertainty about fatherhood and fears of rejection by future partners. While they did not equate masculinity with fertility as strongly as previous studies suggest (Ussher and Perz 2019), the issue still affected their self-image and sense of completeness.

### “My values and perspectives have changed”

Another key finding was a shift in participants’ values and perspectives post-treatment. Despite loss and a diminished sense of self, many developed a deeper appreciation for everyday moments, relationships, and avoiding trivial conflicts. This aligns with research on late effects and post-traumatic growth, which links value shifts to identity reconstruction (Hauken et al. 2019; McDonnell et al. 2018), though few studies have focused specifically on men or YACSs. As participants’ capacities declined, pre-cancer values became harder to maintain. This may be understood as a reduced sense of agency and a stronger emphasis on communal values (Gebauer et al. 2013), helping them realign goals with their new reality and fostering a more coherent self-perception and identity. Here, acceptance emerged as a crucial factor in recovery, aligning with previous studies (Secinti et al. 2019). Difficulty accepting new limitations may prolong adjustment, as clinging to pre-cancer standards can hinder progress and erode self-worth. When goals become unattainable, identity may suffer. Interestingly, participants who experienced fewer life changes acknowledged that identity reconstruction would have been necessarily had their capacity been more severely affected.

### “I have some advice to share”

Participants often felt isolated, echoing findings from mixed-gender YACS studies (Benedict et al. 2018; Hauken and Larsen 2019). This isolation stemmed from falling behind peers in education, work, and relationships due to cancer and its late effects. A lack of understanding from family, friends, and even healthcare professionals can intensify this sense of disconnection (Hauken et al. 2013). Since social validation is vital for identity, being treated differently can threaten one’s self-concept (Shelton et al. 2006).

Survivorship became a central part of participants’ social identity—viewed either positively (“I survived”) or negatively (“I am different from my peers”). Many emphasized the value of connecting with other male YACSs who could relate to their experiences and support their evolving identity. Belonging is a basic human need (Chan et al. 2019). According to Agency and Communion theory, traits like trustworthiness and altruism are key to group cohesion and achieving shared goals (Chan et al. 2019). However, YACSs may be perceived as less agentic—less capable—leading to exclusion (Neuberg and Cottrell 2008), while group membership often favors those seen as competent contributors.

Contrary to research suggesting men are reluctant to share emotions or seek help (Galdas et al. 2005), participants encouraged open dialogue with peers and professionals. Sharing helps reconstruct identity by piecing together life’s fragments (Carlick and Biley 2004). Still, finding other male YACSs can be difficult due to their small numbers.

Participants also advised a gradual return to life post-treatment. Rushing back often created a mismatch between mental readiness and physical capacity, disrupting identity. They encouraged reflection, patience, exploring new roles, and exploring different identities. These findings suggest male YACSs may enter a moratorium phase—reassessing goals, values, and identity considering their changed capacities (Marcia 1966). Despite feeling diminished, participants showed resilience, adapting to their new reality and finding meaning in the process.

### Study’s strengths and limitations

This study offers new insights into how male YACSs experience and cope with identity-related challenges. A key strength is its qualitative design, which amplifies the voices of an under-researched group. Data saturation was achieved, capturing a range of perspectives and circumstances, resulting in a rich and nuanced understanding of identity reconstruction. The transparent and systematic analytical approach further strengthens the study’s credibility.

However, some limitations should be noted. Despite data saturation, certain challenges may have gone unaddressed. Although our sample included YACS with diverse cancer diagnoses and treatment modalities, it may not fully capture the nuanced differences across subgroups—such as those treated with orchidectomy versus chemotherapy or major surgical procedures like limb amputation. Participants who had already processed their experiences may have been more inclined to participate, potentially excluding those still struggling. Additionally, the sample did not include the youngest male survivors, as participants were aged 23–39. Still, the consistency of themes and subthemes, supported by direct quotes, suggests that key aspects of male YACSs’ post-treatment identity were captured. Given the qualitative nature of this study, the findings are not generalizable to the broader population. Instead, they may serve as a foundational basis for future research and to inform the development of clinical practices.

### Implications for clinical practice and recommendations for further research

This study underscores the importance of peer support for YACSs in processing their cancer journey and ongoing challenges. However, male YACSs represent a small fraction of new cancer cases annually, often leading to isolation due to difficulty finding peers with similar experiences. Healthcare professionals should facilitate early support group formation to help male YACSs share experiences and build social networks. Coping with reduced physical, cognitive, or social capacity required participants to acknowledge their losses— akin to a grieving process. Acceptance was key to moving forward, setting new goals, and fostering positive identity development. Based on these findings, early access to professional support is recommended to assist YACSs in transitioning from patient to a redefined self. Further research is warranted to investigate subgroup differences related to cancer diagnosis and treatment, and to better understand how factors such as social support, personal goals, and individual values influence identity reconstruction among male YACSs following cancer. Additionally, the development of a standardized instrument to quantify the frequency and perceived importance of identity-related concerns and themes could provide valuable insights and support future intervention design.

## Conclusion

This study indicates that male YACSs undergo a significant identity transformation following cancer, marked by increased maturity and a shift in values. Limitations in physical, cognitive, and social functioning seem to disrupt their goals, ambitions, and sense of mastery, prompting a move from agentic to more communal qualities.

Acceptance of their new reality and letting go of their pre-cancer identity were identified as key to adaptation. Participants emphasized the importance of speaking with others—especially fellow male YACSs or healthcare professionals—to process emotions and reflect on priorities. Taking time to adjust was seen as essential for changed and matured identity in survivorship. These findings may serve as a foundational basis for future research and to inform the development of clinical practices.

## Supplementary Information

Below is the link to the electronic supplementary material.

Supplementary Material 1

## Data Availability

The datasets generated during and/or analyzed during the current study are available from the corresponding author on reasonable request.
